# Shrink, weighing accuracy, and weighing precision of mineral supplement in five commercial dairies in the Western United States

**DOI:** 10.1093/tas/txab087

**Published:** 2021-05-10

**Authors:** J A Arce-Cordero, S L Archibeque, A P Faciola

**Affiliations:** 1 Department of Animal Sciences, University of Florida, Gainesville, FL 32611, USA; 2 Escuela de Zootecnia, Universidad de Costa Rica, San Jose, Costa Rica; 3 Department of Animal Sciences, Colorado State University, Fort Collins, CO 80521, USA

**Keywords:** feed tracking software, premix, total mixed ration

## Abstract

Shrink, accuracy, and precision of ingredient weighing are critical factors of efficiency in TMR-fed dairy systems. Those factors have been evaluated for major feeds; however, we are not aware of any reports for mineral supplement. Farms commonly mix mineral supplement with other low-inclusion ingredients into a premix which is used later as a single ingredient for TMR formulation. Our objectives were to evaluate shrink, weighing accuracy, and weighing precision of mineral supplement during premix formulation, and variation in concentration of minerals in mineral supplement and TMR, in five large dairies in the Western United States. We used the automated weight-tracking system at each farm to account for all the mineral supplement loaded into the mixing-wagon and collected samples of mineral supplement and TMR from time of mineral supplement delivery at the farm until 100% of it was consumed. Mean, standard deviation and coefficient of variation (CV) for each variable were calculated with SAS 9.4. Average shrink was estimated at 2.0% for mineral supplement during storage and loading, ranging from 0.37% to 3.25%. Mineral supplement weighing deviation from the targeted amount was 1.54% on average for the five dairies with a 95% CV. Mineral composition of mineral supplements averaged 11.3%, 0.27%, and 3.16% for Ca, P, and Mg, and 215, 881, and 1533 ppm for Cu, Mn, and Zn, respectively. Mineral compositions in TMR averaged 0.84%, 0.41%, and 0.37% for Ca, P, and Mg, respectively; and 15.1, 71, and 94.5 ppm for Cu, Mn, and Zn, respectively. The CV of all minerals except Ca, were larger for mineral supplement than TMR, and with the exception of P in mineral supplement, CV of trace minerals were larger than CV values for macro minerals. Our shrink estimates for mineral supplement represent an initial approximation to this issue. Results of our weighing deviation analysis suggest some room for improvement on the precision of weighing mineral supplement at the time when premix is prepared at the farm, which could improve consistency in chemical composition of the premix and consequently reduce the variation (CV values) of mineral concentrations in TMR.

## INTRODUCTION

The American dairy industry has experienced considerable changes over the past decades, when the midpoint herd size increased from 80 to 900 cows between 1987 and 2012 (MacDonald et al., 2016). Consequently, more than 50% of dairy cows in the U.S. are found in large farms (>500 cows). This trend has been more evident in the Western states, where 80% of the cows are found in operations with more than 500 cows (USDA-NASS, 2007). These farms manage cows under confinement and purchase feeds in bulk to prepare customized total mixed rations (TMR) in order to reduce feeding costs (MacDonald et al., 2016). However, they also face unique challenges in terms of maximizing utilization of feeds and nutrients to improve profitability of the business.

Commodity shrink associated with storage, loading, and mixing of feeds can be a determining factor of efficiency in dairy farms (Standaert et al., 1994). Different aspects are expected to affect commodity shrink; including wind, design of storage facilities, and type of feed (Soriano, 2014). Whereas up to 40% shrink is expected for wet byproducts (such as wet distillers grains) stored in three-sided bays; an average 3% to 15% shrink has been reported for grains and dry by products under the same storage conditions, with greater values corresponding to bulky feeds such as cottonseed, wheat bran, and soybean hulls (Kertz, 1998). Given the higher density, nutrient concentration and cost of mineral supplement in comparison to other feeds, it is important to study the shrink of mineral supplement on farms, which may differ from estimates for other types of feeds and has not been previously reported in literature.

Ingredient weighing accuracy and precision are important factors impacting efficiency of TMR-feeding systems and depend—among other things—on the type of feed loaded into the mixing-truck (Trillo et al., 2016). As a strategy to improve distribution of micronutrients in the TMR, large dairies normally prepare a premix with the feeds that have a small level of inclusion in the diet, such as the mineral supplement, buffers, and additives; plus a fraction of the grain from the diet; then this premix is included as a whole new ingredient with a greater level of inclusion in TMR formulation. However, reports of weighing accuracy and precision of mineral supplement into the premix preparation are absent in literature.

For this study we aimed to: 1) estimate shrink of mineral supplement during storage, 2) evaluate weighing accuracy and precision of mineral supplement when loaded into the mixing-wagon for premix preparation, and 3) evaluate variation in mineral concentration of mineral supplement and TMR on five large commercial dairies in the Western U.S. region.

## MATERIALS AND METHODS

Five large dairy farms with an average size between 500 and 2400 cows, located in the Western U.S. region, were chosen for this study. All the dairies were managed with a TMR feeding system and their diets consisted of corn silage-based rations prepared with vertical mixers ranging from 10,900 to 13,600 kg of maximum capacity. Based on samples collected for this study, the average chemical composition of TMR across the five farms evaluated, was as follows: 16.8% to 18.8% CP, 30.3% to 35.7% NDF, 1.57% to 1.60 Mcal/kg NEl, 0.74% to 0.93% Ca, 0.38% to 0.45% P, 0.32% to 0.45% Mg, 14 to 16 ppm Cu, 61 to 84 ppm Mn and 68 to 172 ppm Zn.

The study was conducted in 2015 from June through November, and farms were enrolled based on the following criteria: 1) bulk storage of loose mineral supplement in a three-sided concrete bay under a roof, 2) adequate equipment for TMR preparation (scales, loader, and mixing-wagon), 3) well-established feeding routine (feeding schedule, trained personnel in charge, loading sequence, mixing times and amounts of feeds), 4) use of feed-tracking software allowing to track the weight of mineral supplement from moment of delivery until loaded into the mixing-wagon, and 5) willingness to participate in the study, share feed-tracking system data and allow for collection of samples for chemical analyses.

Both the truck scales and mixing-wagon scales were calibrated before collection of data and samples started in each dairy. All the farms in this study received mineral supplement delivered by a semi-truck and weighed at time of delivery at the truck scales. The mineral supplement storage areas on each farm were emptied and cleaned by the time the mineral load analyzed was delivered, avoiding chances of mixing with residues of a previous load of mineral supplement. Later when almost all the mineral supplement had been incorporated into premix, residual mineral supplement was swept, collected with a shovel and accounted for calculations. In general, feed and mineral supplement management upon delivery was similar across farms and both feed management and diets were kept constant within each farm during the study period.

Among the five farms evaluated, two of them used the EZ feed tracking software (DHI Provo Inc., Provo, UT) and the three other used Feed Watch 7.0 (Valley Agricultural Software Inc., Tulare, CA). At each dairy we tracked a complete load of mineral supplement which averaged 22,426 ± 935 kg (mean ± SD) of as fed product across farms. Daily data of feed-tracking software were collected at each farm from the day of mineral supplement delivery until 100% of the supplement had been used for premix preparation and mixed into TMR. This period was different for each farm, ranging between 10 and 50 days, primarily depending on the size of the dairy. More than one type of TMR was prepared at each dairy; however, for our analysis we focused on the TMR for high producing cows and the mineral supplement used exclusively for this ration.

Samples of TMR were collected daily from the feed bunk throughout the evaluation period at the time of morning and afternoon TMR delivery (two subsamples per day). Approximately 10 kg of TMR were collected by hand along the feed bunk at each feeding time before cows were turned into the pens. Then, the two TMR subsamples collected on the same day were mixed, quartered and composite into one daily sample of 1 kg. Mineral supplement samples were collected with a plastic scoop directly from the pile in the storage area, at the same times when TMR samples were collected. Approximately 500 g were collected at each sampling time, which were combined into a daily composite sample.

Because the total number of days for collection differed among farms (depending on number of days required to consume a complete semi-truck load of mineral supplement at each dairy), then the total number of collection days was divided into 10 equal periods so all the samples within the same period were combined and quartered into a composite sample of either TMR or mineral supplement. Finally, 10 composited samples of TMR and 10 composited samples of mineral supplement from each farm were used for analyses.

Mineral samples were stored at room temperature and TMR samples were stored at −20 °C until analyzed. Samples of TMR were dried in a forced-air oven at 60 °C for 48 h and ground through a 1 mm screen in a Wiley mill (model N°2; Arthur H. Thomas Co., Philadelphia, PA). All samples (TMR and mineral supplement) were analyzed for dry matter at 105 °C (AOAC International, 1990; method 930.15) and concentrations of Ca, Mg, P, Cu, Mn, and Zn determined by inductively coupled plasma mass spectrometry (AOAC International, 2000; method 985.01). Intra-assay CV and inter-assay CV for mineral analyses were ≤8.0% and ≤15.0%, respectively.

Data from the weight-tracking system at each dairy, were exported and processed to estimate the total amount of mineral supplement that was loaded into the mixing-wagon for preparation of premix, as an estimate of utilization during the period analyzed. Shrink was expressed as the difference between the total amount of mineral supplement delivered to the farm minus the total amount—from that same pile—that was loaded into the mixing wagon and therefore accounted for in premix preparation. Wind speed was recorded continuously (at 5-minute intervals) during the period analyzed using a portable Kestrel® meter model 4500 that was installed at each dairy in the mineral supplement storage/loading area. Average speed recorded at the five dairies was 3.6 ± 3.5 km/h (mean ± SD).

Weighing accuracy and precision of mineral supplement loaded into the mixing-wagon during premix preparation, was also analyzed for all the batches of premix that were prepared with the load of mineral supplement that was evaluated at each farm. This was done by comparing the targeted amount of mineral supplement to the amount that was actually loaded into the mixing-wagon. Deviations were expressed on an as fed basis, as a percentage of the targeted amount, negative deviations indicating a smaller than targeted amount of mineral supplement loaded into the mixing-wagon, and the opposite holding true for positive deviations.

Data for shrink estimates, weighing accuracy and precision, and chemical composition were analyzed with the MEANS procedure of SAS 9.4 (SAS Institute Inc., Cary, NC). Means and standard deviations of these variables were calculated individually for each farm.

## RESULTS AND DISCUSSION

Estimates of mineral supplement shrink are shown in Table 2. Amount of mineral supplement delivered at the farms in this study ranged between 21,564 and 23,895 kg, which corresponds in all cases to a semi-truck load. At least part of the variation in weight of mineral supplement load delivered among farms, may be due to different ingredient composition and density among mineral supplement products. As previously mentioned, mineral supplements are custom-made for each farm to be fed at a given level of inclusion (also established for each farm) and to complement the nutrients provided by other feeds in the ration. Therefore, although some core components are normally included in the mineral supplements, their proportions and specific sources vary among mineral supplements, which makes physical and chemical composition unique for each formula.

**Table 1.** Description of commercial dairy farms evaluated in the study

Farm	Herd size^1^	Number of feeders	Evaluation period, d^2^	Premix loads		
				N^3^	Size, kg/load^4^	Inclusion of mineral, %^5^
1	2400	2	10	3	13636	43.9
2	500	2	51	33	9091	7.1
3	1500	2	26	18	12273	9.0
4	1700	2	15	32	9091	7.2
5	2300	2	15	30	10909	6.7

^1^Lactating cows.

^2^Number of days required to consume a batch of mineral supplement equivalent to a semi-truck.

^3^Number of premix loads prepared exclusively with mineral supplement from the evaluated batch.

^4^Average size of premix loads over the study period (as fed basis).

^5^Percent inclusion of mineral supplement on premix loads (as fed basis).

**Table 2.** Shrink estimates of mineral supplement in five commercial dairy farms

Farm	Total amount delivered, kg	Total amount incorporated into premix, kg	Total loss, kg	Estimated shrink, %
1	21,672	21,592	80	0.37
2	22,419	22,282	137	0.61
3	21,564	21,064	500	2.32
4	22,581	21,960	621	2.75
5	23,895	23,118	777	3.25
Mean	22,426	22,003	423	2.0

Shrink of mineral supplement due to storage and handling losses, ranged from 0.37% to 3.25% of the total amount of mineral supplement that was initially delivered, and averaged 2.0% across the five farms. Such a range of shrinkage suggests that different management practices may influence losses of mineral supplement during storage and daily handling. Moreover, mineral supplement shrink observed for the five farms in our study, is close to the lowest values reported for grains and byproducts stored in covered three-sided bays (3% to 15%); where lower values correspond to concentrates and meals, and higher values are associated with brans, pulps, and more fibrous feed sources (Kertz, 1998); indicating that perhaps feeds with higher density, such as mineral supplement, would be less prone to shrink in comparison to other ingredients.

Other reports for dry commodities weighed-in as semi loads and weighed-out in the mixing trucks, indicate shrink values of 2% to 4% (Loy, 2010) and our average estimate was close to the lower end of this range. Although a small percentage, such losses could represent a considerable waste of nutrients given the high nutrient density of mineral supplement in comparison to other feeds. Further research in this area, would provide insights on the implications of such losses, including financial aspects which can be closely related to management of commodities (Standaert et al., 1994). Assessment of different options for storage, loading, and mixing of mineral supplements in dairy diets may provide some guidance on how to minimize losses, as well as costs and benefits associated to those strategies.

An analysis performed in dairy farms of California, revealed that for most of the farms in the study, median of weighing deviations for feeds that were loaded into the mixing-wagon during TMR preparation, ranged from −2% to 2% (Trillo et al., 2016). In our study, we specifically evaluated the weighing deviations for mineral supplement loaded to the mixing-wagon during premix preparation (Table 3), which ranged from 0.3% to 2.8% with a mean value of 1.54% across the five dairies evaluated. Also, during the same period, for the five dairies in our study we observed the following average weighing deviations for major feeds in TMR (data not shown): 0.82% for silage, 0.91% for grain, 6.4% for hay, and 7.1% for wet byproducts; suggesting that perhaps factors associated with storage conditions and physical properties of mineral supplement may favor smaller weighing deviations in comparison to bulky and high-moisture feeds, such as hay and wet byproducts, respectively. Smaller average weighing deviations for inclusion of corn silage and grain in the TMR, than average deviation for inclusion of mineral supplement in the premix, suggest a possible room for improvement in the weighing accuracy of mineral supplement on these farms.

**Table 3.** Weighing accuracy of mineral supplement loaded into the mixing-wagon during premix preparation in five commercial dairy farms

	Mineral supplement inclusion, kg/load^1^		Deviation from target		
Farm	Target	Actual	kg^2^	%^3^	CV
1	5990	6008	18.0	0.3	31
2	648	665	18.1	2.8	131
3	1110	1121	17.8	1.6	109
4	656	670	8.5	1.3	140
5	732	747	12.4	1.7	65

^1^Level of inclusion of mineral supplement per load of premix, expressed on as fed basis.

^2^Expressed as kg of mineral supplement per load of premix (Actual—Target).

^3^Expressed as a percentage of the targeted level of inclusion of mineral supplement.

One factor influencing precision and accuracy while weighing ingredients into the mixing-wagon is the skills of the person driving the loader (Buckmaster, 2009). The busy feeding schedule at the dairies may increase the susceptibility of drivers to either under or over-dose ingredients to save time by reducing trips between the mixing truck and the piles of ingredients. James and Cox (2008) evaluated weighing deviations of major TMR ingredients on 15 dairies in Virginia, finding that mean deviations were larger for primary feeders (in charge of weighing, mixing and delivering TMR at least 75% of the time) in comparison to secondary feeders, suggesting that not only skills of the feeder are important but also that experienced feeders may develop undesirable habits over time. In the case of our study, estimated deviation values correspond to primary feeders and the observed variation across farms may be influenced not only by operator skills but also to by the distance between the mineral supplement storage area in relation to the mixing-wagon (James and Cox, 2008).

Weighing precision of mineral supplement into the mixing-wagon can be analyzed by evaluating the coefficients of variation for the average deviations. As observed in Table 3, on average such deviations had a CV of 95%, indicating a rather large range of variation. A wide range in deviations (expressed as kg per load) has already been reported for major TMR ingredients such as corn silage (James and Cox, 2008); however, expressing deviations as absolute weight units assigns the largest deviations and variations to ingredients with greater levels of inclusion in the TMR, such as corn silage. We avoided this confounding factor by expressing mineral supplement weighing deviations as a percentage of the targeted level of inclusion in the premix. For that same reason, although deviation from the targeted amount of mineral supplement in the premix was a very similar amount (kg) for dairies 1, 2, and 3 (Table 3), the percentage deviation in farm 1 was 5 and 9 times smaller than those observed in farms 3 and 2, respectively.

Another possible factor influencing weighing precision of mineral supplement is the amount loaded to the mixing-wagon during premix preparation. As observed in Table 3, farm 1 with the lowest CV also has the largest level of inclusion of mineral supplement per premix load. In this case, a large level of inclusion of mineral supplement per premix load, reduces the frequency and therefore the total number of premix loads prepared over time (as shown in Table 1), which may have an impact on feeders weighing precision.

Impact of loading inaccuracy is larger when the nutrient concentration of the ingredient greatly differs from that of the ration and is also proportional to the level of inclusion of that ingredient (Buckmaster and Muller, 1994). High concentrations of mineral elements in mineral supplements may be an important reason to improve weighing accuracy and precision of mineral supplements during premix preparation at the farms. In this regard, we evaluated the variation in mineral concentration of both mineral supplements and TMR along the evaluated period in the five dairies of our study (Table 4).

**Table 4.** Variation in concentration of minerals in mineral supplement and TMR over 10 to 50 d in five commercial dairy farms

Feed	Statistics	Farm	DM, %	Macro-minerals, %			Micro-minerals, ppm		
				Ca	P	Mg	Cu	Mn	Zn
Mineral supplement	Mean^1^	1	91.6	3.68	0.56	1.40	61	279	361
		2	96.7	12.1	0.30	3.95	199	885	949
		3	87.6	13.5	0.06	5.68	240	560	1503
		4	94.9	12.1	0.38	2.46	238	1110	1216
		5	89.3	15.3	0.04	2.31	339	1572	3635
		Overall^2^	92.0	11.3	0.27	3.16	215	881	1533
	CV^3^	1	0.82	12.2	5.18	9.17	22.8	14.0	5.97
		2	0.41	5.11	9.31	5.21	17.5	14.2	16.7
		3	3.46	13.2	62.1	44.6	20.2	30.4	23.2
		4	1.47	4.36	5.98	5.74	6.68	5.37	17.5
		5	1.17	2.38	70.0	5.73	26.0	14.7	15.1
		Overall^4^	1.47	7.5	30.5	14.1	18.6	15.7	15.7
TMR	Mean^1^	1	47.1	0.86	0.38	0.45	14.5	65.6	81.4
		2	60.1	0.93	0.45	0.37	15.8	76.5	80.3
		3	48.8	0.89	0.44	0.37	15.9	60.5	70.8
		4	56.8	0.81	0.42	0.33	13.8	67.7	68.1
		5	54.0	0.74	0.38	0.32	15.5	84.4	172
		Overall^2^	53.4	0.84	0.41	0.37	15.1	71.0	94.5
	CV^3^	1	3.54	16.9	5.80	6.84	2.93	5.75	6.17
		2	2.27	13.9	6.13	7.88	7.96	5.77	10.5
		3	2.58	8.80	6.35	11.8	17.5	13.6	11.5
		4	1.44	11.5	3.05	5.49	15.6	13.4	13.3
		5	2.73	17.2	4.29	3.25	11.0	8.29	8.04
		Overall^4^	2.51	13.7	5.10	7.10	11.0	9.40	9.90

^1^Mean values within-farm (calculated from 10 samples per farm).

^2^Mean values across farms.

^3^CV values within-farm.

^4^Average CV values across farms.

Variation in mineral concentration of mineral supplements for the five dairies evaluated show average CV values across farms between 15% and 18% for Cu, Mn, and Zn; and smaller average CV values for macro minerals Ca (7%) and Mg (14%), which may be influenced by the lower concentrations of trace minerals in comparison to Ca and Mg. A similar trend was observed by Rossow and Aly (2013) who found larger CV values for Cu, Mn, and Zn than for macro nutrients in TMR of high producing cows in California dairies. In our study, a high average CV was observed for P (30%) as a result of the very low concentration of P in the mineral supplements of dairies 3 and 5 (0.06 and 0.04% P, respectively) in comparison to the other three farms which supplements averaged 0.41% P. The low P mineral supplements did not include any source of P, then their P concentration was close to 0%; therefore, any small contribution of P from other ingredients in the mineral supplement or from contamination during storage or handling would slightly increase P concentration, having a large impact on the CV. Consequently, a high CV for P may be inflated and not representative of the real variation in P concentration of mineral supplements in the dairies we evaluated. Excluding the two dairies for which mineral supplement is devoid of P, the average CV for concentration of this element in mineral supplement is only 7%.

In regards to within-farm variation, CV values for mineral composition of both mineral supplement and TMR (Table 4) were higher in farm 3 than in the four other farms for all the macro and micro-minerals tested in the mineral supplement and for all the micro-minerals and macro-minerals in TMR, except for Ca. Besides real variation in chemical composition of mineral supplement and TMR, resulting from mixing quality of the mineral supplement and TMR ingredient loading errors, respectively; multiple sources of error, such as sampling error and laboratory analytical errors, may contribute to the variation observed in mineral concentration of mineral supplement and TMR.

Turiello et al. (2018) found the effect of farm as the main component of variance observed in DM concentration of corn silage and TMR at dairy farms, explaining anything between 40% to 92% of the variation in DM concentration. Cherney et al. (2021) found a large week-to-week variation in chemical composition of corn silage and TMR, but a small variation due to sampling across four dairies evaluated. St-Pierre and Weiss (2015) estimated farm and monthly variation as the largest sources of variability in chemical composition of corn silage; while for TMR samples, variation resulting from sampling and lab analyses (together) was one of the main sources for variation across farms, accounting for approximately 40% of total variance. Estimates of within-farm variance by St-Pierre and Weiss (2015) indicated that variation due to sampling and lab analyses accounted for 58% of the observed variation in chemical composition of TMR samples collected in a same farm over a period of 12 consecutive months, showing the importance of this source of variation even when presumably all the samples are collected by the same person analyzed in the same lab.

Concentration of minerals in mineral supplements and its variation, are important because mineral supplements are a significant source of minerals for the animal. However, such impact gets diluted when the mineral supplement is mixed with other ingredients to prepare the premix and further diluted when the premix is mixed with other major feeds during TMR formulation. Average concentrations of minerals in TMR and their variation, for the five dairies evaluated in this study, are shown in Table 4. As expected, concentrations of both macro and trace minerals in TMR are lower than concentrations of the same elements in mineral supplement; and similarly, average CV values for all mineral elements—except Ca—are lower in TMR than in mineral supplement.

Greater variation in %Ca of TMR than in %Ca of mineral supplement, may be a result of the large relative contribution of silage to the total %Ca of TMR. Although silage is not one of the feeds with the highest concentration of Ca, the combination of a modest Ca concentration with a high level of inclusion in the TMR, makes it one of the main contributors of Ca to the TMR. Weighing of ingredients is performed on a wet basis; therefore, some feeds such as silage with lower and more variable DM concentrations, can become important sources of variation in TMR’s chemical composition, such as %Ca of TMR in this particular case.

Sova et al. (2014) evaluated variation in chemical composition of TMR from 24 dairies in Ontario, Canada. They observed CV values of 7.7%, 4.1%, and 5.2% for Ca, P, and Mg, and 9.9%, 10.7%, and 9.2% for Cu, Mn, and Zn, respectively. St-Pierre and Weiss (2015) estimated different components of the variation in nutrient composition of TMR from 47 dairy farms (20 in Ohio and 27 in nine other states); they estimated respective CV values of 4.7%, 3.7%, and 6.5% for Ca, P, and Mg, and 9.1%, 10.8%, and 13.0% for Cu, Mn, and Zn. Variation in mineral concentrations of TMR observed in our study agrees with those reports, showing a similar pattern where CV for trace minerals is approximately twice that of CV observed for macro minerals, with exception of Ca as discussed above.

To the best of our knowledge, our study is the first to evaluate variation in chemical composition of both mineral supplement and TMR simultaneously, showing a greater average variation (higher CV values) in mineral supplement than in TMR mineral composition, and larger CV values for trace minerals than for macro minerals in both mineral supplement and TMR.

Although our study did not intend to evaluate a representative sample of U.S. dairies, our data provide a basis for future studies. Shrink estimates for mineral supplements represent an initial approximation to this issue. In this regard, cost-benefit analysis would be crucial to determine not only the potential reduction of losses resulting from a given management strategy, but also the cost associated to those management strategies. On the other hand, variation in mineral composition of TMR observed in our study was in agreement with values reported in previous studies, and most importantly, our weighing deviation analysis suggests some room for improvement on the precision of weighing mineral supplement at the time when premix is prepared at the farm, which could improve consistency in chemical composition of the premix and consequently reduce the variation (CV values) of mineral concentrations in TMR.

*Conflict of interest statement*. The authors declare no conflict of interest.
